# Analysis and Numerical Simulations of a Stochastic SEIQR Epidemic System with Quarantine-Adjusted Incidence and Imperfect Vaccination

**DOI:** 10.1155/2018/7873902

**Published:** 2018-02-20

**Authors:** Fei Li, Xinzhu Meng, Xinzeng Wang

**Affiliations:** ^1^College of Mathematics and Systems Science, Shandong University of Science and Technology, Qingdao 266590, China; ^2^State Key Laboratory of Mining Disaster Prevention and Control Co-Founded by Shandong Province and the Ministry of Science and Technology, Shandong University of Science and Technology, Qingdao 266590, China

## Abstract

This paper considers a high-dimensional stochastic SEIQR (susceptible-exposed-infected-quarantined-recovered) epidemic model with quarantine-adjusted incidence and the imperfect vaccination. The main aim of this study is to investigate stochastic effects on the SEIQR epidemic model and obtain its thresholds. We first obtain the sufficient condition for extinction of the disease of the stochastic system. Then, by using the theory of Hasminskii and the Lyapunov analysis methods, we show there is a unique stationary distribution of the stochastic system and it has an ergodic property, which means the infectious disease is prevalent. This implies that the stochastic disturbance is conducive to epidemic diseases control. At last, computer numerical simulations are carried out to illustrate our theoretical results.

## 1. Introduction

Mathematical models for differential equations have been widely applied in various fields [1–7]. Specifically, they have had a realistic significance to analyze the dynamical behaviors in the field of mathematical biology [8–17], which obtained some novel results.

In fact, the main meaning of the research of infectious disease dynamics is to make people more comprehensively and deeply understand the epidemic regularity of infectious disease; then more effective control strategies are adopted to provide better theoretical support for the prevention and control of epidemics. To this end, many mathematical biology workers considered more realistic factors in the course of the study, such as population size change, migration, cross infection, and other practical factors. In the course of epidemics and outbreaks of infectious diseases, people always take various measures to control the epidemic in order to minimize the harm of epidemic diseases. Quarantine is one of the important means to prevent and control epidemic diseases; it has been used to control contagious diseases with some success. Specifically, during the severe acute respiratory syndrome (SARS) outbreak in 2002, remarkable results were also achieved. Among them, mathematical models have been used to investigate their impact on the dynamics of infectious diseases under quarantine [18–22], which attracts deep research interest of many mathematicians and biologists. Recently, Hethcote et al. [21] considered an SIQR (susceptible-infected-quarantined-recovered) model with quarantine-adjusted incidence. The system can be expressed as follows:

(1) S˙=Λ−βSIN~−Q−μS,I˙=βSIN~−Q−λ+ε1+γ+μI,Q˙=λI−φ+ε2+μQ,R˙=γI+φQ−μR,

where the total population size is given by $\tilde{N}(t)=S(t)+I(t)+Q(t)+R(t)$, Λ is the inflow rate corresponding to birth and immigration, and *μ* is the outflow rate corresponding to natural death and emigration. Since the quarantine process, using the standard incidence $\left(\beta I/\tilde{N}\right)S$, the contact rate $\beta Q/\tilde{N}$ with the quarantined fraction $Q/\tilde{N}$ does not occur. Hence the standard incidence is replaced by $\beta SI/\left(\tilde{N}-Q\right)$ (it is called quarantine-adjusted incidence); here *β* is the transmission coefficient between susceptible individuals and infected individuals. *λ* is the quarantine rate of infected individuals, *φ* is the recovery rate of quarantined individuals, and *ε*_1_ and *ε*_2_ stand for the rate of disease-related death of infected and quarantined individuals, respectively. *γ* is the recovery rate of infected individuals. Furthermore, all the parameters are positive and the region $\tilde{D}=\left\{(S,I,Q,R)|S\geq 0,\;I\geq 0,\;Q\geq 0,\;R\geq 0,\;S+I+Q+R\leq \Lambda /\mu \right\}$ is a positively invariant set of system (1). In the region $\tilde{D}$, they established the basic reproduction number *R*_0_, which determines disease extinction or permanence, where

(2) $$\begin{array}{c} R_{0}=\frac{\beta }{\lambda +\varepsilon_{1}+\gamma +\mu }. \end{array}$$

Meanwhile, they analyzed the global dynamics of system (1) and derived the equilibria (including the disease-free equilibrium and the endemic equilibrium) and their global stability. In addition, the parameter conditions for the existence of a Hopf bifurcation are obtained.

In the real world, with the development of modern medicine, vaccination has become an important strategy for disease prevention and control in addition to quarantine, and numerous scholars have investigated the effect of vaccination on disease [23–30]. For another, many infectious diseases incubate inside the hosts for a period of time before becoming infectious, so it is very meaningful to consider the effect of the incubation period. Motivated by the aforementioned work, this paper considers an SEIQR (susceptible-exposed-infected-quarantined-recovered) epidemic model with imperfect vaccination, which is described by the following system:

(3) S˙=Λ−1−δβSIN−Q−δ1−pβSIN−Q−δp+μS,E˙=1−δβSIN−Q+δ1−pβSIN−Q−α+μE,I˙=αE−λ+ε1+γ+μI,Q˙=λI−φ+ε2+μQ,R˙=δpS+γI+φQ−μR,

where the total population size is given by *N*(*t*) = *S*(*t*) + *E*(*t*) + *I*(*t*) + *Q*(*t*) + *R*(*t*), *δ*  (0 ≤ *δ* < 1) is the vaccine coverage rate, *p*  (0 ≤ *p* ≤ 1) is the vaccine efficacy, and *α* is the rate at which the exposed individuals become infected individuals. Other parameters are the same as in system (1). Now we assume that all the parameters are positive constants here except that *δ*, *p* are nonnegative constants. Clearly, the region *D* = {(*S*, *E*, *I*, *Q*, *R*)∣*S* ≥ 0, *E* ≥ 0, *I* ≥ 0, *Q* ≥ 0, *R* ≥ 0, *S* + *E* + *I* + *Q* + *R* ≤ Λ/*μ*} is a positively invariant set of system (3). For system (3), the basic reproduction number is

(4) $$\begin{array}{c} R_{1}=\frac{\mu \left(1-\delta p\right)\beta \alpha }{\left(\delta p+\mu \right)\left(\alpha +\mu \right)\left(\lambda +\varepsilon_{1}+\gamma +\mu \right)} \end{array}$$

and it has the following properties:

1. When *R*_1_ ≤ 1 holds, system (3) has a unique disease-free equilibrium *E*_0_ = (*S*_0_, 0,0, 0, *R*_0_) = (Λ/(*δp* + *μ*), 0,0, 0, *δp*Λ/*μ*(*δp* + *μ*)) which is globally asymptotically stable in the region *D*. That means the epidemic diseases will die out and the total individuals will become the susceptible and recovered individuals.
2. When *R*_1_ > 1 holds, system (3) has a unique globally asymptotically stable positive equilibrium *E*^*∗*^ = (*S*^*∗*^, *E*^*∗*^, *I*^*∗*^, *Q*^*∗*^, *R*^*∗*^) in the region *D*, which means the epidemic diseases will persist.

In the natural world, deterministic model is not enough to describe the species activities. Sometimes, the species activities may be disturbed by uncertain environmental noises. Consequently, some parameters should be stochastic [31–40]. There is no denying that this phenomenon is ubiquitous in the ecosystem. Therefore numerous scholars have introduced the effect of stochastic perturbation on diseases [41–50]. To the best of our knowledge, the research on global dynamics of the stochastic SEIQR epidemic model with imperfect vaccination is not too much yet. In this paper, to make system (3) more reasonable and realistic, we assume the environmental noise is directly proportional to *S*(*t*), *E*(*t*), *I*(*t*), *Q*(*t*), and *R*(*t*). Then corresponding to system (3), a stochastic version can be reached by

(5) dS=Λ−1−δβSIN−Q−δ1−pβSIN−Q−δp+μSdt+σ1SdB1t,dE=1−δβSIN−Q+δ1−pβSIN−Q−α+μEdt+σ2EdB2t,dI=αE−λ+ε1+γ+μIdt+σ3IdB3t,dQ=λI−φ+ε2+μQdt+σ4QdB4t,dR=δpS+γI+φQ−μRdt+σ5RdB5t,

where *B*_*i*_(*t*)  (*i* = 1,2, 3,4, 5) is the mutually independent standard Wiener process with *B*_*i*_(0) = 0 a.s. *σ*_*i*_(*t*)  (*i* = 1,2, 3,4, 5) is a continuous and bounded function for any *t* ≥ 0 and *σ*_*i*_^2^(*t*)  (*i* = 1,2, 3,4, 5) are the intensities of Wiener processes.

In this paper, we are mainly concerned with two interesting problems as follows:

(P1) Under what parameter conditions, will the disease die out?
(P2) Under what conditions, will system (5) have a unique ergodic stationary distribution?

Throughout this paper, let (*Ω*, *ℱ*, {*ℱ*}_*t*≥0_, *ℙ*) be a complete probability space with a filtration {*ℱ*_*t*_}_*t*≥0_ satisfying the usual conditions (i.e., it is increasing and right continuous while *ℱ*_0_ contains all *ℙ*-null sets). Further *B*_*i*_(*t*)  (*i* = 1,2, 3,4, 5) is defined on the complete probability space.

For simplicity and convenience, we introduce the following notations:

1. *ℝ*_+_ = [0, +*∞*), *ℝ*_+_^5^ = {*x* = (*x*_1_, *x*_2_, *x*_3_, *x*_4_, *x*_5_) ∈ *ℝ*^5^ : *x*_*i*_ > 0  (*i* = 1,2, 3,4, 5)}.
2. For an integrable function *x*(*t*)∈[0, +*∞*), 〈*x*(*t*)〉 = (1/*t*)∫_0_^*t*^*x*(*r*)d*r*.
3. *a*∧*b* = min⁡{*a*, *b*}, *a*∨*b* = max⁡{*a*, *b*}.

## 2. Global Positive Solution

To investigate the dynamical behaviors of a population system, we first concern the global existence and positivity of the solutions of system (5).

**Lemma 1 .**

For any given initial value (*S*(0), *E*(0), *I*(0), *Q*(0), *R*(0)) ∈ *ℝ*_+_^5^, system (5) has a unique positive local solution (*S*(*t*), *E*(*t*), *I*(*t*), *Q*(*t*), *R*(*t*)) for *t* ∈ [−*ω*, *τ*_*e*_), where *τ*_*e*_ is the explosion time [51].

**Theorem 2 .**

For any given initial value (*S*(0), *E*(0), *I*(0), *Q*(0), *R*(0)) ∈ *ℝ*_+_^5^, system (5) has a unique positive solution (*S*(*t*), *E*(*t*), *I*(*t*), *Q*(*t*), *R*(*t*)) ∈ *ℝ*_+_^5^ on *t* ≥ 0 a.s.

**Proof**

The following proof is divided into two parts.

*Part I*. By Lemma 1, it is easy to see that system (5) has a unique positive local solution (*S*(*t*), *E*(*t*), *I*(*t*), *Q*(*t*), *R*(*t*)) for any given initial value (*S*(0), *E*(0), *I*(0), *Q*(0), *R*(0)) ∈ *ℝ*_+_^5^.

*Part II*. Now we prove that the positive solution is global, that is, *τ*_*e*_ = *∞* a.s. Let *k*_0_ ≥ 0 be sufficiently large such that *S*(0), *E*(0), *I*(0), *Q*(0), and *R*(0) all lie in [1/*k*_0_, *k*_0_]. For each integer *k* ≥ *k*_0_, let us define the stopping time as follows:

(6) τk=inf⁡t∈−ω,τe:St∉1k,k,Et∉1k,k,It∉1k,k,Qt∉1k,k  or  Rt∉1k,k,

where we define inf⁡*∅* = *∞* (*∅* represents the empty set). Evidently, *τ*_*k*_ is strictly increasing when *k* → *∞*. Let *τ*_*∞*_ = lim_*k*→*∞*_*τ*_*k*_; thus *τ*_*∞*_ ≤ *τ*_*e*_ a.s. So we just need to show that *τ*_*∞*_ = *∞* a.s. If *τ*_*∞*_ = *∞* is untrue, then there exist two constants *T* > 0 and *ς* ∈ (0,1) such that *ℙ*{*τ*_*∞*_ ≤ *T*} > *ς*. Thus there exists *k*_1_ ≥ *k*_0_  (*k*_1_ ∈ *N*_+_) such that

(7) $$\begin{array}{c} P\left\{\;\tau_{k}\leq T\;\right\}\geq \varsigma ,\;k\geq k_{1}. \end{array}$$

Define a *C*^2^-function $\hat{V}:{\mathbb{R}}_{+}^{5}\to {\mathbb{R}}_{+}$ by

(8) V^S,E,I,Q,R=S−1−ln⁡S+E−1−ln⁡E+I−1−ln⁡I+Q−1−ln⁡Q+R−1−ln⁡R.

Applying Itô's formula and system (5), we have

(9) dV^=LV^dt+σ1S−1dB1t+σ2E−1dB2t+σ3I−1dB3t+σ4Q−1dB4t+σ5R−1dB5t,

where

(10) LV^=1−1S·Λ−1−δβSIN−Q−δ1−pβSIN−Q−δp+μS+1−1E·1−δβSIN−Q+δ1−pβSIN−Q−α+μE+1−1IαE−λ+ε1+γ+μI+1−1Q·λI−φ+ε2+μQ+1−1R·δpS+γI+φQ−μR+12σ12+σ22+σ32+σ42+σ52=Λ+δp+α+λ+γ+φ+ε1+ε2+5μ+1−δpβIS+E+I+R−μS+E+I+Q+R−ε1I−ε1Q−ΛS−1−δpβSIES+E+I+R−αEI−λIQ−δpSR−γIR−φQR+12σ12+σ22+σ32+σ42+σ52≤Λ+δp+α+λ+γ+φ+ε1+ε2+5μ+1−δpβ+12σ12+σ22+σ32+σ42+σ52=M0,

and here *M*_0_ is a positive constant. Hence

(11) dV^≤M0dt+σ1S−1dB1t+σ2E−1dB2t+σ3I−1dB3t+σ4Q−1dB4t+σ5R−1dB5t.

Integrating both sides of (11) from 0 to *τ*_*k*_∧*T* and then taking the expectation, we have

(12) EV^Sτk∧T,Eτk∧T,Iτk∧T,Qτk∧T,Rτk∧T≤V^S0,E0,I0,Q0,R0+E∫0τk∧TM0dt≤V^S0,E0,I0,Q0,R0+M0T.

Set *Ω*_*k*_ = {*τ*_*k*_ ≤ *T*}, *k* ≥ *k*_1_ and by (7) we can get that *P*(*Ω*_*k*_) ≥ *ς*. Notice that, for every *ω* ∈ *Ω*_*k*_, there exists *S*(*τ*_*k*_, *ω*), *E*(*τ*_*k*_, *ω*), *I*(*τ*_*k*_, *ω*), *Q*(*τ*_*k*_, *ω*), or *R*(*τ*_*k*_, *ω*) which equals either 1/*k* or *k*. Thus

(13) V^Sτk,ω,Eτk,ω,Iτk,ω,Qτk,ω,Rτk,ω≥1k−1−ln⁡1k∧k−1−ln⁡k.

By virtue of (12) and (13), one has

(14) V^S0,E0,I0,Q0,R0+M0T≥E1ΩkωV^Sτk,ω,Eτk,ω,Iτk,ω,Qτk,ω,Rτk,ω≥ς1k−1−ln⁡1k∧k−1−ln⁡k,

and here 1_*Ω*_*k*_(*ω*)_ is the indicator function of *Ω*_*k*_(*ω*). Let *k* → *∞*, which implies

(15) $$\begin{array}{c} \infty >\hat{V}\left(\;S\left(\;0\;\right),E\left(\;0\;\right),I\left(\;0\;\right),Q\left(\;0\;\right),R\left(\;0\;\right)\;\right)+M_{0}T=\infty \end{array}$$

is a contradiction. Obviously, we get that *τ*_*∞*_ = *∞*. This completes the proof of Theorem 2.

## 3. Extinction

In this section, we mainly explore the parameter conditions for extinction of the disease in system (5). Before proving the main results, we first give a useful lemma as follows.

**Lemma 3 .**

For any given initial value (*S*(0), *E*(0), *I*(0), *Q*(0), *R*(0)) ∈ *ℝ*_+_^5^, the solution (*S*(*t*), *E*(*t*), *I*(*t*), *Q*(*t*), *R*(*t*)) of the system (5) has the following properties:

(16) limt→∞⁡Stt=0,limt→∞⁡Ett=0,limt→∞⁡Itt=0,limt→∞⁡Qtt=0,limt→∞⁡Rtt=0a.s.

Furthermore, when *μ* > (1/2)(*σ*_1_^2^∨*σ*_2_^2^∨*σ*_3_^2^∨*σ*_4_^2^∨*σ*_5_^2^) holds, then

(17) limt→∞⁡1t∫0tSrdB1r=0,limt→∞⁡1t∫0tErdB2r=0,limt→∞⁡1t∫0tIrdB3r=0,limt→∞⁡1t∫0tQrdB4r=0,limt→∞⁡1t∫0tRrdB5r=0a.s.

**Proof**

The proof of Lemma 3 is similar to [25, 41]; thus we omit it here.

**Theorem 4 .**

Let *μ* > (1/2)(*σ*_1_^2^∨*σ*_2_^2^∨*σ*_3_^2^∨*σ*_4_^2^∨*σ*_5_^2^). For any given initial value (*S*(0), *E*(0), *I*(0), *Q*(0), *R*(0)) ∈ *ℝ*_+_^5^, if

(18) R~∗≔2α1−δpβα+μλ+ε1+γ+μ+σ32/2α+μ2∧α2σ22/2<1

holds, then

(19) $$\begin{array}{c} \underset{t\to \infty }{\lim}E\left(\;t\;\right)=\underset{t\to \infty }{\lim}I\left(\;t\;\right)=\underset{t\to \infty }{\lim}Q\left(\;t\;\right)=0\;\text{a.s.} \end{array}$$

Moreover,

(20) limt→∞⁡S=Λδp+μ=S0,limt→∞⁡R=δpΛμδp+μ=R0a.s.

**Proof**

Define a differentiable function *V*_0_ by

(21) $$\begin{array}{c} V_{0}=\ln\left[\;\alpha E\left(\;t\;\right)+\left(\;\alpha +\mu \;\right)I\left(\;t\;\right)\;\right]. \end{array}$$

From Itô's formula and system (5), we have

(22) dV0=α1−δpβSI/S+E+I+R−α+μλ+ε1+γ+μIαE+α+μI−α2σ22E2+α+μ2σ32I22αE+α+μI2dt+ασ2EαE+α+μIdB2t+α+μσ3IαE+α+μIdB3t≤α1−δpβα+μ−λ+ε1+γ+μ+σ32/2α+μ2I2+α2σ22/2E2αE+α+μI2dt+ασ2EαE+α+μIdB2t+α+μσ3IαE+α+μIdB3t≤α1−δpβα+μ−λ+ε1+γ+μ+σ32/2α+μ2∧α2σ22/22α+μ2dt+ασ2EαE+α+μIdB2t+α+μσ3IαE+α+μIdB3t.

Integrating from 0 to *t* and dividing by *t* on both sides of (22), we have

(23) ln⁡αEt+α+μItt≤α1−δpβα+μ−λ+ε1+γ+μ+σ32/2α+μ2∧α2σ22/22α+μ2+ln⁡αE0+α+μI0t+ασ2t∫0tErαEr+α+μIrdB2r+α+μσ3t∫0tIrαEr+α+μIrdB3r.

Making use of Lemma 3, we have

(24) lim supt→∞⁡ln⁡αEt+α+μItt≤α1−δpβα+μ−λ+ε1+γ+μ+σ32/2α+μ2∧α2σ22/22α+μ2<0a.s.,

which shows that

(25) limt→∞⁡Et=0,limt→∞⁡It=0a.s.

From the fourth equation of system (5), it is easy to get that

(26) $$\begin{array}{c} \underset{t\to \infty }{lim}Q\left(\;t\;\right)=0\;\text{a.s.} \end{array}$$

Moreover, integrating from 0 to *t* and dividing by *t* on both sides of the first equation of system (5) yield

(27) St−S0t=Λ−1−δpβSIN−Q−δp+μS+σ1t∫0tSrdB1r,

and considering (25), (26), and Lemma 3, it then follows that

(28) $$\begin{array}{c} \underset{t\to \infty }{\lim}\langle \;S\;\rangle =\frac{\Lambda }{\delta p+\mu }=S_{0}\;\text{a.s.} \end{array}$$

Similarly, we also get

(29) $$\begin{array}{c} \underset{t\to \infty }{\lim}\langle \;R\;\rangle =\frac{\delta p\Lambda }{\mu \left(\delta p+\mu \right)}=R_{0}\;\text{a.s.} \end{array}$$

The proof of Theorem 4 is complete.

## 4. Stationary Distribution and Ergodicity

Ergodicity is a significant property in a population system. Recently, it attracts deep research interest of numerous scholars [52, 53]. In this section, based on the theory of Hasminskii et al. [54] and the Lyapunov analysis methods, we study the conditions of the existence of an ergodic stationary distribution.

Assume *X*(*t*) as a time-homogeneous Markov process in *𝔼*_*n*_ ⊂ *ℝ*^*n*^, which is described by the stochastic differential equation

(30) $$\begin{array}{c} dX\left(\;t\;\right)=b\left(\;X\;\right)dt+\overset{n}{\underset{\eta =1}{\sum }}\sigma_{\eta }\left(\;X\;\right)dB_{\eta }\left(t\right), \end{array}$$

and here *𝔼*_*n*_ stands for a *n*-dimensional Euclidean space. The diffusion matrix takes the following form:

(31) $$\begin{array}{c} \tilde{A}\left(\;x\;\right)=\left(\;a_{ij}\left(\;x\;\right)\;\right),\;a_{ij}\left(x\right)=\overset{n}{\underset{\eta =1}{\sum }}\sigma_{\eta }^{i}\left(x\right)\sigma_{\eta }^{j}\left(x\right). \end{array}$$

**Assumption 5 .**

Assume that there is a bounded domain *U* ⊂ *𝔼*_*n*_ with regular boundary Γ such that $\bar{U}\subset {𝔼}_{n}\;\;(\bar{U}\;\;is\;\;the\;\;closure\;\;of\;\;U)$, satisfying the following properties:

- In the domain *U* and some neighborhood thereof, the smallest eigenvalue of the diffusion matrix $\tilde{A}(x)$ is bounded away from zero.
- If *x* ∈ *𝔼*_*n*_∖*U*, the mean time *τ* at which a path issuing from *x* reaches the set *U* is finite, and sup_*x*∈Θ_*𝔼*_*x*_*τ* < *∞* for every compact subset Θ ⊂ *𝔼*_*n*_.

**Lemma 6 (see [54]).**

When Assumption 5 holds, then the Markov process *X*(*t*) has a stationary distribution *π*(·). Furthermore, when *f*(·) is a function integrable with respect to the measure *π*, then

(32) $$\begin{array}{c} P_{x}\left\{\;\underset{T\to \infty }{\lim}\frac{1}{T}\overset{T}{\underset{0}{\int }}f\left(\;X\left(\;t\;\right)\;\right)dt=\int_{E_{n}}f\left(\;x\;\right)\pi \left(\;dx\;\right)\;\right\}=1 \end{array}$$

for all *x* ∈ *𝔼*_*n*_.

**Remark 7 .**

To demonstrate Assumption 5(i) [55], it suffices to demonstrate that *F* is uniformly elliptical in any bounded domain *H*; here

(33) $$\begin{array}{c} Fu=b\left(\;x\;\right)u_{x}+\frac{1}{2}\text{trace}\left(\;A\left(\;x\;\right)u_{xx}\;\right); \end{array}$$

namely, there exists a positive number *Z* such that

(34) $$\begin{array}{c} \overset{n}{\underset{i,j=1}{\sum }}a_{ij}\left(\;x\;\right)\xi_{i}\xi_{j}\geq Z{\left|\;\xi \;\right|}^{2},\;x\in \bar{H},\;\xi \in R^{n}. \end{array}$$

To verify Assumption 5(ii) [56], it suffices to demonstrate that there exist some neighborhood *U* and a nonnegative *C*^2^-function *V* such that ∀*x* ∈ *𝔼*_*n*_∖*U*, *LV*(*x*) < 0.

Using Lemma 6, we can get the following main results.

**Theorem 8 .**

For any given initial value (*S*(0), *E*(0), *I*(0), *Q*(0), *R*(0)) ∈ *ℝ*_+_^5^. If

(35) R∗≔μ1−δpβαδp+μ+σ12/2α+μ+σ22/2λ+ε1+γ+μ+σ32/2>1

holds, then system (5) has a unique stationary distribution *π*(·) and it has ergodic property.

**Proof**

Define a *C*^2^-function $\bar{V}:{\mathbb{R}}_{+}^{5}\to \mathbb{R}$ by

(36) V¯S,E,I,Q,R=ΥS+E+I+Q+R−a1ln⁡S−a2ln⁡E−a3ln⁡I+1ϱ+1S+E+I+Q+Rϱ+1−ln⁡S−ln⁡E−ln⁡Q−ln⁡R+S+E+I+Q+R≔ΥV1+V2+V3+V4+V5+V6+V7,

and here *ϱ* and *a*_*i*_  (*i* = 1,2, 3) are positive constants satisfying the following conditions:

(37) 0<ϱ<2μσ12∨σ22∨σ32∨σ42∨σ52,a1=Λδp+μ+σ12/2,a2=Λα+μ+σ22/2,a3=Λλ+ε1+γ+μ+σ32/2,

and we take *Υ* > 0 large enough such that

(38) $$\begin{array}{c} -\Upsilon \phi +M\leq -2; \end{array}$$

here

(39) ϕ≔4ΛR∗1/4−1,M≔Λ+Γ+δp+α+φ+ε2+4μ+σ122+σ222+σ422+σ522.

Obviously,

(40) $$\begin{array}{c} \underset{w\to ,\left(S,E,I,Q,R\right)\in R_{+}^{5}\setminus U_{w}}{\lim\;\inf}\bar{V}\left(\;S,E,I,Q,R\;\right)=\infty , \end{array}$$

and here *U*_*w*_ = (1/*w*, *w*) × (1/*w*, *w*) × (1/*w*, *w*) × (1/*w*, *w*) × (1/*w*, *w*). Since $\bar{V}(S,E,I,Q,R)$ is a continuous function, then there exists a unique point $\left(\underline{S^{*}},\underline{E^{*}},\underline{I^{*}},\underline{Q^{*}},\underline{R^{*}}\right)$ in *ℝ*_+_^5^ which is the minimum point of $\bar{V}(S,E,I,Q,R)$. Therefore let us construct a positive-definite *C*^2^-function *V*: *ℝ*_+_^5^ → *ℝ*_+_ by

(41) VS,E,I,Q,R=V¯S,E,I,Q,R−V¯S∗_,E∗_,I∗_,Q∗_,R∗_.

From Itô's formula, we have

(42) LV1=−μS+E+I+Q+R−a1ΛS−a21−δβSIEN−Q−a2δ1−pβSIEN−Q−a3αEI+a11−δβIN−Q+a1δ1−pβIN−Q+Λ+a1δp+μ+σ122+a2α+μ+σ222+a3λ+ε1+γ+μ+σ322−ε1I−ε2Q≤−μS+E+I+Q+R−a1ΛS−a21−δpβSIES+E+I+Q+R−a3αEI+a11−δpβIS+E+I+R+Λ+a1δp+μ+σ122+a2α+μ+σ222+a3λ+ε1+γ+μ+σ322≤−4a1a2a3Λμ1−δpβα1/4+a11−δpβIS+E+I+R+4Λ=−4Λμ1−δpβαδp+μ+σ12/2α+μ+σ22/2λ+ε1+γ+μ+σ32/21/4−1+a11−δpβIS+E+I+R=−4ΛR∗1/4−1+a11−δpβIS+E+I+R=−ϕ+a11−δpβIS+E+I+R.

Similarly,

(43) LV2=S+E+I+Q+Rϱ·Λ−μS+E+I+Q+R−ε1I−ε2Q+ϱS+E+I+Q+Rϱ−12×σ12S2+σ22E2+σ32I2+σ42Q2+σ52R2≤ΛS+E+I+Q+Rϱ−μ−ϱ2σ12∨σ22∨σ32∨σ42∨σ52·S+E+I+Q+Rϱ+1≤Γ−12μ−ϱ2σ12∨σ22∨σ32∨σ42∨σ52·S+E+I+Q+Rϱ+1≤Γ−12μ−ϱ2σ12∨σ22∨σ32∨σ42∨σ52·Sϱ+1+Eϱ+1+Iϱ+1+Qϱ+1+Rϱ+1,

and here

(44) Γ=supS,E,I,Q,R∈R+5⁡ΛS+E+I+Q+Rϱ−12μ−ϱ2σ12∨σ22∨σ32∨σ42∨σ52×S+E+I+Q+Rϱ+1<∞.

We also have

(45) LV3=−ΛS+1−δpβIS+E+I+R+δp+μ+σ122,LV4≤−1−δpβSIES+E+I+Q+R+α+μ+σ222,LV5=−λIQ+φ+ε2+μ+σ422,LV6=−δpS+γI+φQR+μ+σ522,LV7≤Λ−μS+E+I+Q+R.

Therefore,

(46) LV≤−Υϕ+Υa11−δpβIS+E+I+R+1−δpβIS+E+I+R−12μ−ϱ2σ12∨σ22∨σ32∨σ42∨σ52×Sϱ+1+Eϱ+1+Iϱ+1+Qϱ+1+Rϱ+1−ΛS−1−δpβSIES+E+I+Q+R−μS+E+I+Q+R−λIQ−δpS+γI+φQR+Λ+Γ+δp+α+φ+ε2+4μ+σ122+σ222+σ422+σ522≤−Υϕ+Υa1+11−δpβIS+E+I+R−12μ−ϱ2σ12∨σ22∨σ32∨σ42∨σ52·Sϱ+1+Eϱ+1+Iϱ+1+Qϱ+1+Rϱ+1−ΛS−2μ1−δpβSIE1/2−λIQ−δpS+γI+φQR+M,

where *M* = Λ + Γ + *δp* + *α* + *φ* + *ε*_2_ + 4*μ* + *σ*_1_^2^/2 + *σ*_2_^2^/2 + *σ*_4_^2^/2 + *σ*_5_^2^/2.

Next let us consider the following compact subset *D*:

(47) D=ϵ≤S≤1ϵ,ϵ4≤E≤1ϵ4,ϵ2≤I≤1ϵ2,ϵ3≤Q≤1ϵ3,ϵ4≤R≤1ϵ4,

and here *ϵ* is a sufficiently small constant satisfying the following conditions:

(48) −Λϵ+Υa1+11−δpβ+M≤−1,

(49) −2μ1−δpβϵ1/2+Υa1+11−δpβ+M≤−1,

(50) −Υϕ+Υa1+11−δpβϵ+M≤−1,

(51) −λϵ+Υa1+11−δpβ+M≤−1,

(52) −δpϵ3−γϵ2−φϵ+Υa1+11−δpβ+M≤−1,

(53) −12μ−ϱ2σ12∨σ22∨σ32∨σ42∨σ521ϵϱ+1+Υa1+11−δpβ+M≤−1,

(54) −12μ−ϱ2σ12∨σ22∨σ32∨σ42∨σ521ϵ4ϱ+1+Υa1+11−δpβ+M≤−1,

(55) −12μ−ϱ2σ12∨σ22∨σ32∨σ42∨σ521ϵ2ϱ+1+Υa1+11−δpβ+M≤−1,

(56) −12μ−ϱ2σ12∨σ22∨σ32∨σ42∨σ521ϵ3ϱ+1+Υa1+11−δpβ+M≤−1.

Then

(57) R+5∖D=D1∪D2∪D3∪D4∪D5∪D6∪D7∪D8∪D9∪D10,

with

(58) D1=S,E,I,Q,R∈R+5 ∣ 0<S<ϵ,D2=S,E,I,Q,R∈R+5 ∣ S≥ϵ,I≥ϵ2,0<E<ϵ4,D3=S,E,I,Q,R∈R+5 ∣ S≥ϵ,0<I<ϵ2,D4=S,E,I,Q,R∈R+5 ∣ I≥ϵ2,0<Q<ϵ3,D5=S,E,I,Q,R∈R+5 ∣ S≥ϵ,I≥ϵ2,Q≥ϵ3,0<R<ϵ4,D6=S,E,I,Q,R∈R+5 ∣ S>1ϵ,D7=S,E,I,Q,R∈R+5 ∣ E>1ϵ4,D8=S,E,I,Q,R∈R+5 ∣ I>1ϵ2,D9=S,E,I,Q,R∈R+5 ∣ Q>1ϵ3,D10=S,E,I,Q,R∈R+5 ∣ R>1ϵ4.

Now we analyze the negativity of *ℒV* for any (S, *E*, *I*, *Q*, *R*) ∈ *ℝ*_+_^5^∖*D*.

*Case I*. If (*S*, *I*, *Q*, *R*) ∈ *D*_1_, (46) and (48) derive that

(59) LV≤−ΛS+Υa1+11−δpβIS+E+I+R+M≤−Λϵ+Υa1+11−δpβ+M≤−1.

*Case II*. If (*S*, *I*, *Q*, *R*) ∈ *D*_2_, (46) and (49) imply that

(60) LV≤−2μ1−δpβSIE1/2+Υa1+11−δpβIS+E+I+R+M≤−2μ1−δpβϵ1/2+Υa1+11−δpβ+M≤−1.

*Case III*. If (*S*, *I*, *Q*, *R*) ∈ *D*_3_, it follows from (46) and (50) that

(61) LV≤−Υϕ+Υa1+11−δpβIS+E+I+R+M≤−Υϕ+Υa1+11−δpβIS+M≤−Υϕ+Υa1+11−δpβϵ+M≤−1.

*Case IV*. If (*S*, *I*, *Q*, *R*) ∈ *D*_4_, (46) and (51) yield that

(62) LV≤−λIQ+Υa1+11−δpβIS+E+I+R+M≤−λϵ+Υa1+11−δpβ+M≤−1.

*Case V*. If (*S*, *I*, *Q*, *R*) ∈ *D*_5_, it follows from (46) and (52) that

(63) LV≤−δpS+γI+φQR+Υa1+11−δpβIS+E+I+R+M≤−δpϵ3−γϵ2−φϵ+Υa1+11−δpβ+M≤−1.

*Case VI*. If (*S*, *I*, *Q*, *R*) ∈ *D*_6_, (46) and (53) lead to

(64) LV≤−12μ−ϱ2σ12∨σ22∨σ32∨σ42∨σ52Sϱ+1+Υa1+11−δpβIS+E+I+R+M≤−12μ−ϱ2σ12∨σ22∨σ32∨σ42∨σ521ϵϱ+1+Υa1+11−δpβ+M≤−1.

*Case VII*. If (*S*, *I*, *Q*, *R*) ∈ *D*_7_, (46) and (54) derive that

(65) LV≤−12μ−ϱ2σ12∨σ22∨σ32∨σ42∨σ52Eϱ+1+Υa1+11−δpβIS+E+I+R+M≤−12μ−ϱ2σ12∨σ22∨σ32∨σ42∨σ521ϵ4ϱ+1+Υa1+11−δpβ+M≤−1.

*Case VIII*. If (*S*, *I*, *Q*, *R*) ∈ *D*_8_, it follows from (46) and (55) that

(66) LV≤−12μ−ϱ2σ12∨σ22∨σ32∨σ42∨σ52Iϱ+1+Υa1+11−δpβIS+E+I+R+M≤−12μ−ϱ2σ12∨σ22∨σ32∨σ42∨σ521ϵ2ϱ+1+Υa1+11−δpβ+M≤−1.

*Case IX*. If (*S*, *I*, *Q*, *R*) ∈ *D*_9_, (46) and (56) derive that

(67) LV≤−12μ−ϱ2σ12∨σ22∨σ32∨σ42∨σ52Qϱ+1+Υa1+11−δpβIS+E+I+R+M≤−12μ−ϱ2σ12∨σ22∨σ32∨σ42∨σ521ϵ3ϱ+1+Υa1+11−δpβ+M≤−1.

*Case X*. If (*S*, *I*, *Q*, *R*) ∈ *D*_10_, it follows from (46) and (54) that

(68) LV≤−12μ−ϱ2σ12∨σ22∨σ32∨σ42∨σ52Rϱ+1+Υa1+11−δpβIS+E+I+R+M≤−12μ−ϱ2σ12∨σ22∨σ32∨σ42∨σ521ϵ4ϱ+1+Υa1+11−δpβ+M≤−1.

Clearly, from the discussion of the above ten cases, one sees that, for a sufficiently small *ϵ*,

(69) $$\begin{array}{c} LV\leq -1\;\forall \left(S,E,I,Q,R\right)\in R_{+}^{5}\setminus D, \end{array}$$

which shows that Assumption 5(ii) is satisfied. In addition, the diffusion matrix of system (5) takes the following form:

(70) $$\begin{array}{c} \tilde{A}=\left(\begin{array}{ccccc} \sigma_{1}^{2}S^{2} & 0 & 0 & 0 & 0\\ 0 & \sigma_{2}^{2}E^{2} & 0 & 0 & 0\\ 0 & 0 & \sigma_{3}^{2}I^{2} & 0 & 0\\ 0 & 0 & 0 & \sigma_{4}^{2}Q^{2} & 0\\ 0 & 0 & 0 & 0 & \sigma_{5}^{2}R^{2} \end{array}\right). \end{array}$$

There exists a positive number

(71) $$\begin{array}{c} Z=\underset{\left(S,E,I,Q,R\right)\in \bar{D}}{\min}\left\{\;\sigma_{1}^{2}S^{2},\sigma_{2}^{2}E^{2},\sigma_{3}^{2}I^{2},\sigma_{4}^{2}Q^{2},\sigma_{5}^{2}R^{2}\;\right\} \end{array}$$

such that

(72) ∑i,j=15aijξiξj=σ12S2ξ12+σ22E2ξ22+σ32I2ξ32+σ42Q2ξ42+σ52R2ξ52≥Zξ2,S,E,I,Q,R∈D¯,  ξ∈R5,

which shows that Assumption 5(i) is satisfied. Consequently, system (5) has a unique stationary distribution *π*(·) and it has ergodic property. The proof of Theorem 8 is complete.

**Remark 9 .**

From Theorem 8, we see that if *R*^*∗*^ > 1 holds, then system (5) has a unique ergodic stationary distribution. It is worthwhile noting that if *σ*_*i*_ = 0  (*i* = 1,2, 3,4, 5), the expression of *R*^*∗*^ coincides with the basic reproduction number *R*_1_ of system (3). This shows that we generalize the results of system (3). For another, this theorem also shows that the disease can resist a small environmental noise to maintain the original persistence.

## 5. Conclusions and Simulations

This paper studies the stochastic SEIQR epidemic model with quarantine-adjusted incidence and imperfect vaccination and obtains two thresholds which govern the extinction and the spread of the epidemic disease. Firstly, the existence of a unique positive solution of system (5) with any positive initial value is proved. Then, from Theorems 4 and 8, the sufficient conditions for extinction of the disease and existence of ergodic stationary distribution of the stochastic system are derived by using the theory of Hasminskii and the Lyapunov analysis methods, which means the infectious disease is prevalent. This implies that the stochastic disturbance is conducive to epidemic diseases control. Now we summarize the main conclusions as follows:

- When *μ* > (1/2)(*σ*_1_^2^∨*σ*_2_^2^∨*σ*_3_^2^∨*σ*_4_^2^∨*σ*_5_^2^) and ${\tilde{R}}^{*}=\left(2\alpha (1-\delta p)\beta (\alpha +\mu )\right)/\left(\left(\lambda +\varepsilon_{1}+\gamma +\mu +\sigma_{3}^{2}/2\right)(\alpha +\mu {)}^{2}\wedge \left(\alpha^{2}\sigma_{2}^{2}/2\right)\right)<1$ hold, then the infected individuals go to extinction almost surely.
- When *R*^*∗*^ = (*μ*(1 − *δp*)*βα*)/((*δp* + *μ* + *σ*_1_^2^/2)(*α* + *μ* + *σ*_2_^2^/2)(*λ* + *ε*_1_ + *γ* + *μ* + *σ*_3_^2^/2)) > 1 holds, then system (5) has a unique stationary distribution *π*(·) and it has ergodic property.

To illustrate the results of the above theorems, we next carry out some numerical simulations by the Matlab software. Let us consider the following discretization equations of system (5):

(73) Sk+1=Sk+Λ−1−δβSkIkSk+Ek+Ik+Rk−δ1−pβSkIkSk+Ek+Ik+Rk−δp+μSkΔt+σ1SkΔtζ1,k+σ122Skζ1,k2−1Δt,Ek+1=Ek+1−δβSkIkSk+Ek+Ik+Rk+δ1−pβSkIkSk+Ek+Ik+Rk−α+μEkΔt+σ2EkΔtζ2,k+σ222Ekζ2,k2−1·Δt,Ik+1=Ik+αEk−λ+ε1+γ+μIkΔt+σ3IkΔtζ3,k+σ322Ikζ3,k2−1Δt,Qk+1=Qk+λIk−φ+ε2+μQkΔt+σ4QkΔtζ4,k+σ422Qkζ4,k2−1Δt,Rk+1=Rk+δpSk+γIk+φQk−μRkΔt+σ5RkΔtζ5,k+σ522Rkζ5,k2−1Δt,

and here *ζ*_*i*,*k*_  (*i* = 1,2, 3,4, 5; *k* = 1,2,…, *n*) stands for *N*(0,1) distributed independent random variables and time increment Δ*t* > 0.

**Figure 1:**
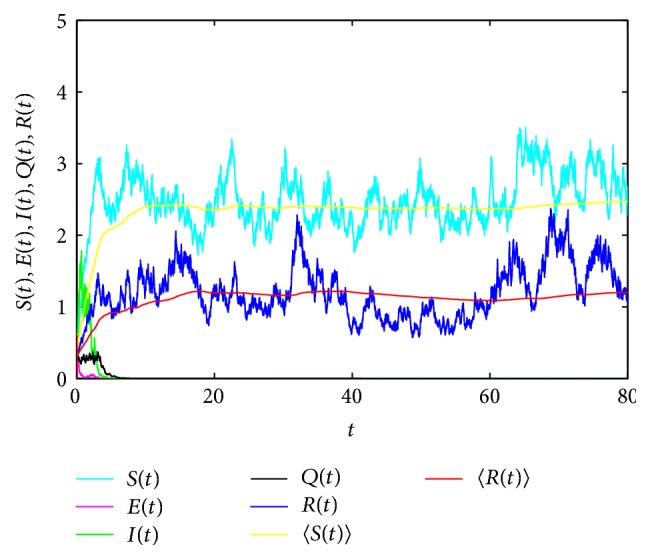
Time sequence diagram of system (5) for extinction of the disease.

In Figure 1, take *S*(0) = *E*(0) = *I*(0) = *Q*(0) = *R*(0) = 0.3, Λ = 2, *μ* = 0.55, *β* = 0.25, *γ* = 0.2, *α* = 2.5, *δ* = 0.5, *p* = 0.5, *ε*_1_ = 0.1, *ε*_2_ = 0.1, *λ* = 0.18, *φ* = 0.15, *σ*_1_ = 0.15, *σ*_2_ = 1, *σ*_3_ = 1, *σ*_4_ = 0.5, *σ*_5_ = 0.25, and Δ*t* = 0.01. Then

(74) μ=0.55>12σ12∨σ22∨σ32∨σ42∨σ52=0.5,R~∗=2α1−δpβα+μλ+ε1+γ+μ+σ32/2α+μ2∧α2σ22/2=0.9150<1

satisfy the conditions in Theorem 4; we can obtain that the exposed, infected, and quarantined individuals go to extinction almost surely. Moreover,

(75) limt→∞⁡S=Λδp+μ=2.5,limt→∞⁡R=δpΛμδp+μ=1.1364a.s.

Obviously, Figure 1 supports our results of Theorem 4.

**Figure 2:**
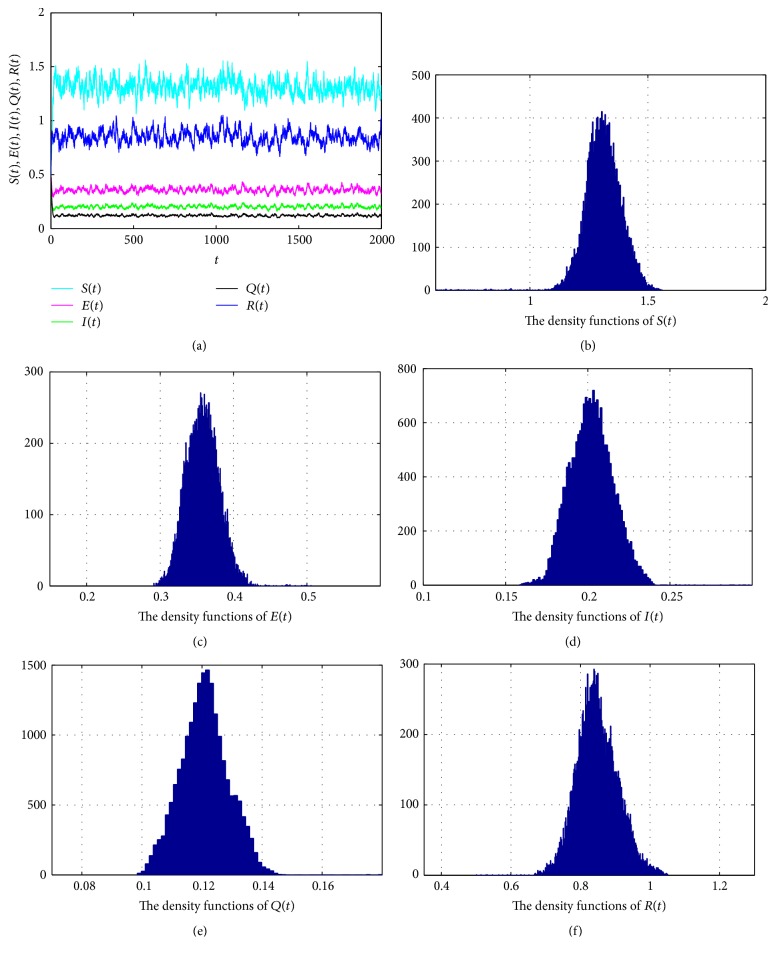
(a) represents the solutions of system (5); (b)–(f) stand for the density functions of *S*(*t*), *E*(*t*), *I*(*t*), *Q*(*t*), and *R*(*t*), respectively.

In Figure 2, take *S*(0) = *E*(0) = *I*(0) = *Q*(0) = *R*(0) = 0.5, Λ = 0.3, *μ* = 0.1, *β* = 1.5, *γ* = 0.2, *α* = 0.3, *δ* = 0.1, *p* = 0.2, *ε*_1_ = 0.05, *ε*_2_ = 0.05, *λ* = 0.18, *φ* = 0.15, *σ*_1_ = *σ*_2_ = *σ*_3_ = *σ*_4_ = *σ*_5_ = 0.03, and Δ*t* = 0.01. Then

(76) R∗=μ1−δpβαδp+μ+σ12/2α+μ+σ22/2λ+ε1+γ+μ+σ32/2=1.7236>1

satisfies the condition in Theorem 8; we can obtain that system (5) has a unique stationary distribution *π*(·) and it has ergodic property. Figure 2 shows that the solution of system (5) swings up and down in a small neighborhood. According to the density functions in Figures 2(b)–2(f), we can see that there exists a stationary distribution. As expected, Figure 2 confirms our results of Theorem 8.

**Figure 3:**
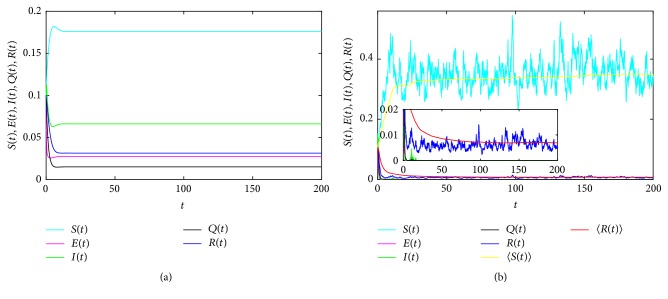
Time sequence diagram of system (5) for persistence and extinction of the disease.

In Figure 3, take *S*(0) = *E*(0) = *I*(0) = *Q*(0) = *R*(0) = 0.1, Λ = 0.2, *μ* = 0.55, *β* = 2.65, *γ* = 0.2, *α* = 2.5, *δ* = 0.1, *p* = 0.1, *ε*_1_ = 0.1, *ε*_2_ = 0.1, *λ* = 0.18, *φ* = 0.15, and Δ*t* = 0.01.

In Figure 3(a), take *σ*_*i*_ = 0  (*i* = 1,2, 3,4, 5); then

(77) $$\begin{array}{c} R_{1}=\frac{\mu \left(1-\delta p\right)\beta \alpha }{\left(\delta p+\mu \right)\left(\alpha +\mu \right)\left(\lambda +\varepsilon_{1}+\gamma +\mu \right)}=2.0505>1, \end{array}$$

which means the disease will persist. As expected, Figure 3(a) shows the disease persists in real life.

Synchronously, in Figure 3(b), take *σ*_1_ = 0.15, *σ*_2_ = 1, *σ*_3_ = 1, *σ*_4_ = 0.5, *σ*_5_ = 0.25. Obviously, Figure 3(b) shows the exposed, infected, and quarantined individuals go to extinction and we can get that the permanent disease of system (3) can die out under stochastic effects. This implies that the stochastic disturbance is conducive to epidemic diseases control.
